# Male-Biased Predation and Its Effect on Paternity Skew and Life History in a Population of Common Brushtail Possums (*Trichosurus vulpecula*)

**DOI:** 10.1371/journal.pone.0111746

**Published:** 2014-11-05

**Authors:** Jane L. DeGabriel, Ben D. Moore, William J. Foley, Christopher N. Johnson

**Affiliations:** 1 Research School of Biology, Australian National University, Canberra, Australian Capital Territory, Australia; 2 School of Marine and Tropical Biology, James Cook University, Townsville, Queensland, Australia; 3 Hawkesbury Institute for the Environment, University of Western Sydney, Richmond, New South Wales, Australia; 4 School of Biological Sciences, University of Tasmania, Hobart, Tasmania, Australia; University of Missouri, United States of America

## Abstract

Differences in predation risk may exert strong selective pressures on life history strategies of populations. We investigated the potential for predation to shape male mating strategies in an arboreal folivore, the common brushtail possum (*Trichosurus vulpecula* Kerr). We predicted that possums in a tropical population exposed to high natural levels of predation would grow faster and reproduce earlier compared to those in temperate populations with lower predation. We trapped a population of possums in eucalypt woodland in northern Australia each month to measure life history traits and used microsatellites to genotype all individuals and assign paternity to all offspring. We observed very high levels of male-biased predation, with almost 60% of marked male possums being eaten by pythons, presumably as a result of their greater mobility due to mate-searching. Male reproductive success was also highly skewed, with younger, larger males fathering significantly more offspring. This result contrasts with previous studies of temperate populations experiencing low levels of predation, where older males were larger and the most reproductively successful. Our results suggest that in populations exposed to high levels of predation, male possums invest in increased growth earlier in life, in order to maximise their mating potential. This strategy is feasible because predation limits competition from older males and means that delaying reproduction carries a risk of failing to reproduce at all. Our results show that life histories are variable traits that can match regional predation environments in mammal species with widespread distributions.

## Introduction

The life history strategies of mammals show remarkable inter- and intra-specific diversity, which may be shaped by several factors, such as resource availability, predation risk and population density [1]–[3]. Promislow and Harvey [4] proposed that mortality is the best predictor of variation in life history traits in mammals, hypothesizing that species and populations with higher rates of mortality should exhibit a faster fecundity schedule and breed at an earlier age, compared to those with lower mortality. Thus, mortality risk is likely to impose a trade-off between strategies to maximize either mating opportunities or survivorship.

The mating strategies of males are also shaped by the spatial and temporal distribution of females, as this determines their ability to defend mates, or to attract females via resource provision [5], [6]. In polygynous mammals, males often range more widely, and are more active due to mate-searching, than are females [5]. In contrast, the home range sizes and distribution of females are more likely to be related to the availability of resources required to optimise their fitness and reproductive success [5]. Thus, in populations where resources are abundant, female home ranges should be small, allowing males to overlap many females at once [7].

A likely consequence of larger home range sizes and greater dispersal distances in males is an increased risk of predation, which imposes a “survival cost” on reproductive effort [8]–[10]. Studies of several mammal species have demonstrated a positive relationship between mobility and predation, resulting in a skew in mortality towards the more mobile sex, which is usually more pronounced during the breeding season [8]. For example, Daly et al. [11] found that male kangaroo rats (*Dipodomys merriami*) were more mobile than were females prior to the breeding season, resulting in higher rates of predation. Similarly, Tecot et al. [12] demonstrated male-biased predation on a population of sifakas (*Propithecus edwardsi*), but only in older age classes, in which males continued to disperse after females had adopted stable home-ranges. Increased mobility has also been linked with higher rates of predation in voles; however this is complicated by the finding that males tend to be more vulnerable to avian predators, whereas females are more susceptible to predation by weasels (*Mustela* sp.) [13].

In populations under high predation pressure, sex-biases in predation risk may have profound effects on key life history traits and social structure, if the vulnerable sex adopts a “live fast, die young” strategy [4]. Thus, where there is a trade-off between reproduction and predator avoidance, the degree of risk-taking behaviour should be inversely related to future reproductive opportunities [9]. In polygynous populations with low death rates of males, younger and smaller individuals are likely to have limited probability of mating success, and thus, the most efficient strategy for a male would be to avoid risk-taking and invest in growth, in order to maximize mating opportunities later in life when a large size has been reached. However, in populations experiencing high predation pressure, males have both the opportunity to mate earlier, as a result of reduced competition from older males, as well as an imperative to do so, because delaying reproduction carries the risk of missing the opportunity to mate at all. Consequently, we would predict a peak in mating success among younger, rather than older males.

Here, we investigated the potential for high rates of predation to shape male mating strategies in a population of common brushtail possums, *Trichosurus vulpecula* Kerr. *T. vulpecula* is a medium-sized arboreal folivorous marsupial (body mass 1500–4500 g), which shows high inter-population variability in body size, demography, diet and habitat [14]. Females produce a single offspring each year, usually in the autumn, although some individuals breed twice per year [14]. Previous studies of native Australian and introduced New Zealand populations have reported a polygynous mating system, with highly skewed male reproductive success [15], [16]. Clinchy et al. [16] found positive relationships between both body size and age, and reproductive success in an Australian population, but Taylor et al. [15] found no effects of these variables in a New Zealand population. A study of tropical *T. vulpecula* on Magnetic Island in north Queensland also found effects of age and size on male reproductive success [17], however in contrast to the study of Clinchy et al. [16], it was younger, rather than older males that were largest and the most reproductively successful.

We used molecular techniques to quantify male reproductive success in a population of *T*. *vulpecula* exposed to high rates of predation by carpet pythons (*Morelia spilota variegata* Gray) in eucalypt woodland in the dry tropics of northern Australia. The observed rate of predation on this tropical population was much higher than that reported in populations from southern Australia [16], and from New Zealand [15]; there are no vertebrate predators of possums in New Zealand, and fewer large reptile predators in southern than in northern Australia. Thus, we predicted that male possums in our population would grow faster and reproduce at a younger age than did possums from populations with lower natural rates of predation.

## Methods

All work complied with the Australian Code of Practice for the Care and Use of Animals for Scientific Purposes and was approved by the James Cook University Animal Ethics Committee (approval number A886_04). It adhered to the conditions of Queensland National Parks and Wildlife Service research permit WISP01170503. We worked in approximately 50 ha of open eucalypt woodland at Tabletop Station, Hervey Range (19°23′S, 146° 27′E) from October 2004 - December 2006. We trapped adult *T*. *vulpecula* at the site at approximately six-week intervals using standard wire cage traps (researcher cat trap, 30 cm×30 cm×60 cm; Mascot Wire Works, Sydney) baited with a mixture of peanut butter, oats and flavoring essence, and collected morphological and demographic data as described in DeGabriel et al. [18]. We calculated an average body mass for each individual from all measurements recorded over each year. During the study, we caught 75 independent adult and subadult *T*. *vulpecula* (49 males and 26 females). We also recorded 73 births (37 males, 32 females and 4 unknown sex) and 16 of these offspring (6 males and 9 females) were subsequently recaptured as independent sub-adults. We determined the age of adult possums according to the tooth-wear index described by Winter [19].

### Home range analysis

We fitted collar-mounted single-stage radio transmitters (Sirtrack Wildlife Tracking Solutions, Havelock North, New Zealand; 6 g) to 20 adult female (91% of all adult females caught between November 2004 and August 2005) and 15 adult male possums (58% of all adult males caught between November 2004 and May 2005). To determine individual home ranges, we radio-tracked possums over 51 nights, between January-April 2005, October 2005 and February-April 2006 as described in DeGabriel et al. [18]. We calculated the home ranges of all possums with ≥20 fixes (*n* = 24; 17 females, 7 males) using 90% minimum convex polygons (MCP), with the Animal Movement Extension in ArcView 3.2 [20].

We mapped the trapping locations of all possums that were caught ≥7 times and calculated their 100% trap-revealed home ranges using MCP. We calculated the center of both the trap-revealed and the radio-tracking revealed ranges for 24 possums to compare whether using trap records alone could provide a reasonable estimate of a possum’s use of the habitat. We then combined these data to re-calculate 100% MCPs for 21 males (81% of all caught) and 21 females (95% of all caught; mean number of locations per animal = 32.8, range = 4–78), and the degree of overlap with other individuals.

### Quantification of predation

On each night, we radio-tracked each possum until we were able to observe it in the canopy or on the ground, or until we determined that the signal was emanating from a python or a python den, such as a hollow log. All pythons located in this way were immobile and exhibited an unmistakable bulge, indicating that they had eaten a possum. In all cases where predation was known or suspected, we tracked the signal during the daytime to recover the collars and confirm consumption after digestion was complete.

We used a Cox proportional hazards model to test the effect of sex on the proportional hazard of predation for all possums that were radio-collared, and for whom status (alive or dead) was known for each month of the study. This is a semi-parametric model, which makes no assumptions about the form of the hazard function h_(t)_, but assumes a parametric form for the effects of the predictor (sex). The model was implemented from the “survival” library in R [21], using the Efron approximation to the partial log-likelihood because of tied events (predation events that were recorded simultaneously) in the data set.

### DNA extraction and microsatellite analysis

We collected a small sample of ear tissue from all possums, including young, and stored them in 100% ethanol at −20°C. We extracted DNA using the salting-out method of Sunnucks and Hales [22] and quantified the concentration using a NanoDrop ND-1000 spectrophotometer (NanoDrop Technologies, Wilmington, DE, USA), before diluting all samples to a standard concentration of 50 ng.µl^−1^.

We genotyped all adults and all pouch young (PY) born between October 2004 and May 2006 at six polymorphic microsatellite loci using primers developed for *T. vulpecula* (Tv16, Tv19, Tv27, Tv53, Tv58 and Tv64) [23]. We performed polymerase chain reaction (PCR) amplifications in 25 µl volumes, consisting of 1 × NH_4_ reaction buffer (Bioline Aust. Pty. Ltd., Alexandria, NSW), 2% bovine serum albumin, 1 unit of *Taq* polymerase (Bioline Aust.) and 1 µL DNA template (approximately 50 ng), made up to volume with sterile distilled water. Concentrations of MgCl_2_, dATP, dCTP, dGTP and dTTP and each primer are given in Table 1. We used a thermal profile consisting of an initial denaturation step of 94°C for 3 min, followed by 35 cycles of 30 s at 94°C, 45 s at annealing temperature of 60°C, 45 s at 72°C (90 s for Tv19 and Tv53), with a final extension step of 72°C for 5 min. To reduce non-specific amplification, we used a touchdown program in the cycling conditions for Tv16 and Tv27, decreasing the annealing temperature in a step-wise manner (Table 1).

**Table 1:** Details of the 6 microsatellite loci and PCR conditions used.

Locus	Fluorescent marker	Annealing temp °C	Allele size range (bp)	MgCl_2_ Conc. (mM)	dNTP Conc. (mM)	Primer Conc. (µM)
Tv16	HEX	65-60 touchdown	114–146	2	0.3	0.2
Tv19	TET	60	254–294	1.2	0.3	0.4
Tv27	HEX	60-55 touchdown	163–193	1.3	0.3	0.5
Tv53	FAM	60	222–272	1	0.3	0.4
Tv58	TET	60	124–168	2	0.2	0.4
Tv64	FAM	60	138–199	1	0.3	0.4

We purified the PCR products using microCLEAN (Microzone Limited, Haywards Heath, UK). Capillary electrophoresis was performed on pooled PCR products using a MegaBACE autosequencer (Amersham Biosciences, Piscataway NJ, USA) at the James Cook University Genetic Analysis Facility. We calculated allele sizes using the MegaBACE Fragment Profiler software (ver. 1.2; Amersham Biosciences, 2003).

### Genetic diversity

We calculated observed (H_o_) and expected (H_e_) heterozygosity of the markers, likely frequency of null alleles, and the average probability of exclusion given one known parent using the parentage analysis program CERVUS 2.0 [24]. We tested for deviation from Hardy-Weinberg expected genotypic proportions (HWE P) using the program GENEPOP, ver. 3.1 [25], both for the whole population and for males and females separately. We also conducted pairwise comparisons of markers to test for independence of genotypes (linkage equilibrium), using a Bonferroni-corrected α value for multiple comparisons [26].

### Analysis of paternity

We considered all adult males as candidate fathers, including juveniles and sub-adults caught in 2004–05 for 2006 births, but excluding radio-collared males after their known date of death. Because all offspring were sampled either when in their mother’s pouch or carried on her back, there was no doubt as to their mother’s identity. We used CERVUS 2.0 [24] to calculate the logarithm of the odds (LOD) scores for identifying the most likely father of each offspring with 99% confidence, according to the criteria applied by Taylor et al. [15].

### Determinants of male reproductive success

We investigated relationships between age, size and reproductive success in tropical male possums. We used linear mixed models with “individual possum” as a random term to test the effects of age, sex and the interaction between age and sex on possum body mass. We then modeled the relationship between age and size for each sex separately using a polynomial function.

We used a *t*-test to determine whether breeding males were heavier than non-breeding males, and a logistic regression model to determine the relationship between male body mass and reproductive success, which we calculated as the number of paternities as a percentage of the total number of offspring produced by females with home ranges overlapping those of the target male. We also used a logistic regression model to determine the relationship between the number of females that a male had access to, as determined by home range overlap and his reproductive success.

We used the same approach to determine the effects of age on reproductive success, but considered data from 2005 only, to avoid problems of considering the same individuals across multiple age classes.

## Results

### Home range analysis

Analyzing the home ranges of individuals from trapping records was a reasonably good indicator of their location as determined by radio-tracking, based on the distance between the center of the range calculated by either method (mean distance ± s.d. = 56.4±37 m, range = 2.8–152.3 m, *n* = 23). The mean home range size of males was 3.9±1.4 ha (mean ± s.d.) and for females 2.1±1.2 ha. On average, individual male home ranges overlapped with those of five known females (range = 2–10 females) as well as with other males. Females did not usually overlap with each other. Only two females showed a large degree of overlap, and given that they did not have mismatching alleles at any locus, we concluded that they were related, probably a mother-daughter pair.

### Quantification of predation

**Figure 1:**
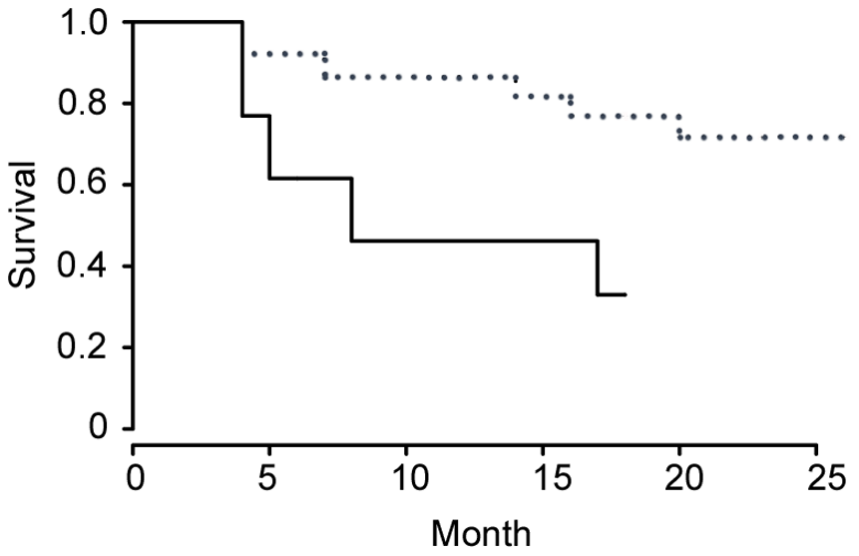
Likelihood of surviving python predation for radio-collared male and female possums. Cox proportional hazards model showing the likelihood of surviving python predation for radio-collared male (solid line) and female (dashed line) possums in each month of the study between October 2004 and November 2006.

We observed a high rate of predation by pythons, strongly biased towards male possums. Radio-tracking revealed that 60% of collared adult males (9 out of 15 individuals) were eaten by pythons between October 2004 and March 2006, compared to only 20% of collared females (4 out of 20 individuals) between October 2004 and November 2006. In addition to the radio-tracking data, we witnessed one incidence of a python ambushing and eating a non-collared male possum. Although other predators such as dingoes (*Canis dingo*), lace monitors (*Varanus varius*) and rufous owls (*Ninox rufa*) were present at the site, only one radio-collared possum (a female) was killed by an owl during the study. Our Cox proportional hazards model revealed that the risk of python predation was 3.88 times (95% confidence interval: 1.17–12.8) greater for male than for female possums (likelihood ratio test on 1 d.f. = 5.74, *P* = 0.019; Fig. 1).

### Genetic diversity

No locus had null alleles at a frequency greater than 0.05, and, after correcting for multiple comparisons, we found no significant linkage disequilibrium between any of the 15 pairwise locus combinations. Considering the population as a whole, we found a significant heterozygote deficit from Hardy-Weinberg genotypic proportions for the locus Tv64 (*P*<0.001), but when we considered the sexes separately, this deficit was significant only in males (*P*<0.001). No deviations from Hardy-Weinberg proportions were evident at any other loci. Genetic diversity was high, with an average of 18.17 alleles per locus, and average H_e_ of 0.897 (Table 2). The combined average exclusion probability given one known parent was >0.999 (Table 2).

**Table 2:** Values for genetic variability measures for the 6 microsatellite loci.

Locus	# alleles	H_o_	H_e_	HWE *P*	Freq. Null alleles	*P* excl.
Tv16	14	0.903	0.888	0.5237	−0.0115	0.771
Tv19	17	0.889	0.889	0.0222	−0.0047	0.773
Tv27	13	0.932	0.912	0.8176	−0.0128	0.813
Tv53	19	0.923	0.906	0.3223	−0.0150	0.805
Tv58	16	0.846	0.845	0.2073	−0.0056	0.707
Tv64	30	0.897	0.942	0.0000	+0.0214	0.876

H_o_ = observed heterozygosity; H_e_ = expected heterozygosity; HWE *P* = Hardy-Weinberg expected genotypic proportions; *P* excl. = probability of exclusion.

### Analysis of paternity

We genotyped 50 offspring at all six loci and one offspring at five loci. There were no mismatches between any offspring and mother pairs. We assigned paternity to 43 (84.3%) of the 51 offspring genotyped with 99% confidence. One male mismatched the mother-offspring pair at locus Tv64, consistent with a possible null allele, and it was considered the most likely father under the criteria used [15]. The remaining seven offspring mismatched the most similar known male at 2 or 3 loci and we deemed them to have unknown paternity (*n* = 7). Six of the seven offspring with unknown fathers had mothers with home ranges on the perimeter of the trapping grid, so it is probable that the fathers were not caught and sampled.

**Figure 2:**
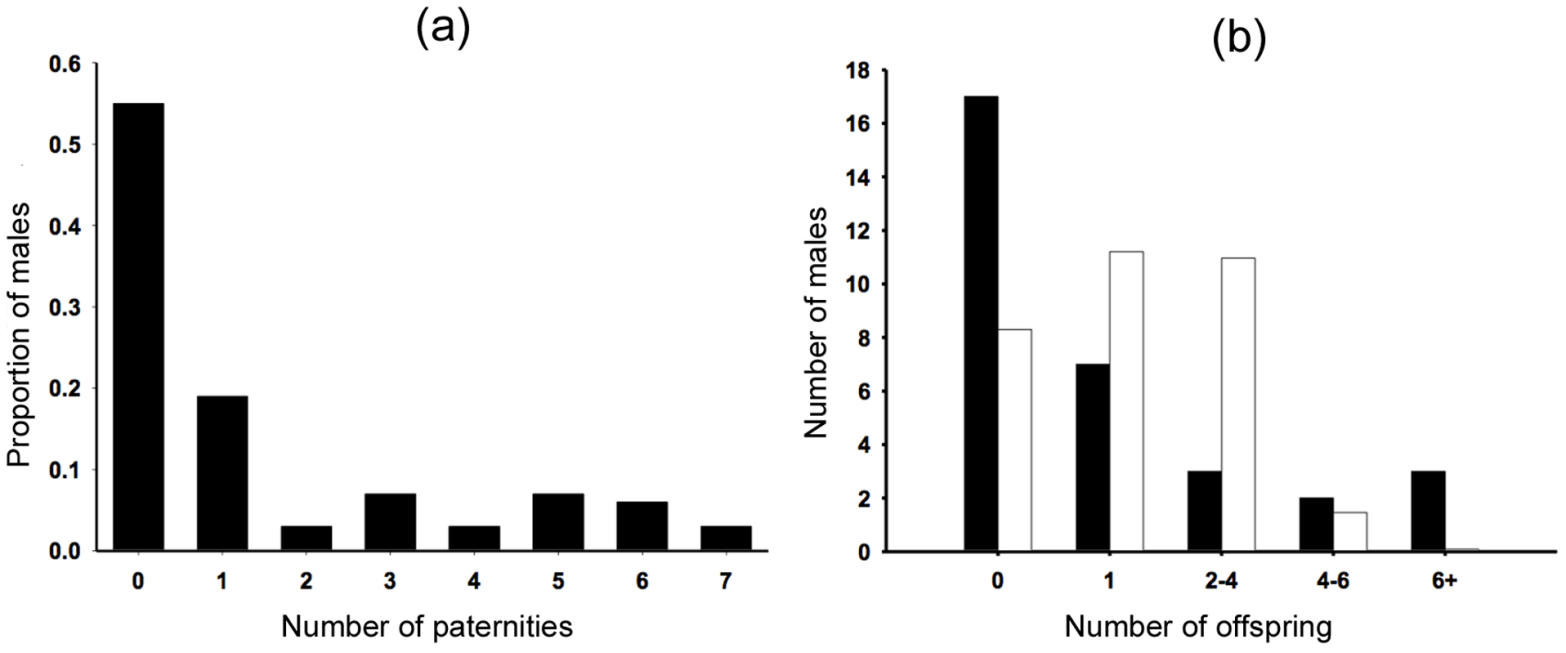
Distribution of paternities amongst all males and skew in number of offspring fathered. (**a**) Histogram showing the distribution of paternities among candidate males, expressed as a proportion of the total number of candidates and (**b**) histogram comparing the observed numbers of males fathering different numbers of offspring (black bars) compared to the number in each group expected from random, as predicted from a Poisson distribution (white bars).

Reproductive output in males was highly skewed. Of the 31 candidate males, 16 (51.6%) were not assigned any paternities in our sample, while five individuals fathered 65% of the genotyped offspring (Fig. 2). The distribution of paternities differed significantly from random, as predicted from a Poisson distribution (χ^2^ = 10.66, *P* = 0.005, d.f. = 2; Fig. 2). Seven males produced offspring with more than one female and three of these produced offspring with four individual females, the highest number observed. The remaining four males that sired multiple offspring sired young with three females. In contrast, 29% of the females who produced multiple offspring (*n* = 17) mated exclusively with a single male, while 75% of females that produced three or more offspring (*n* = 12) mated at least twice with the same male. As a result, 33.3% of the 39 sibling pair relationships genotyped were full-siblings.

### Determinants of male reproductive success

**Figure 3:**
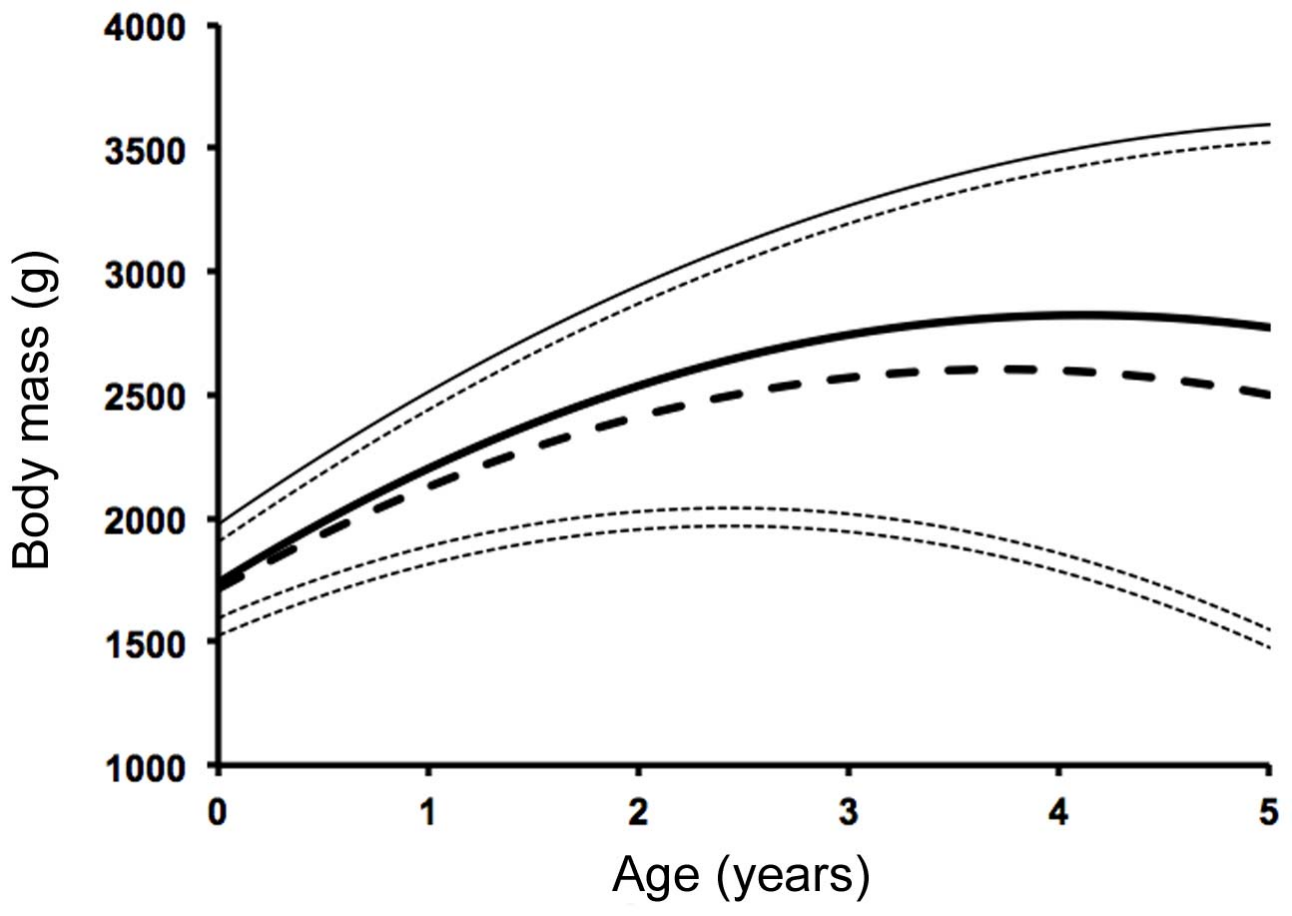
Relationship between age and body mass for male and female possums. Polynomial regression model showing the relationship between age and body mass for female (dashed lines) and male (solid lines) possums. Heavy lines indicate the regression model, lighter lines indicate 95% confidence intervals.

Age had a significant effect on body mass when included as either a linear (Wald = 213.11, *P*<0.001, *n* = 394) or quadratic (Wald = 381.1, *P*<0.001, *n* = 394) term. There was no effect of sex on body mass, but the interaction between sex and age was significant (Wald = 16.71, *P*<0.001, *n* = 394). The relationship between age and size for each sex modeled by a polynomial function is shown in Fig. 3.

**Figure 4:**
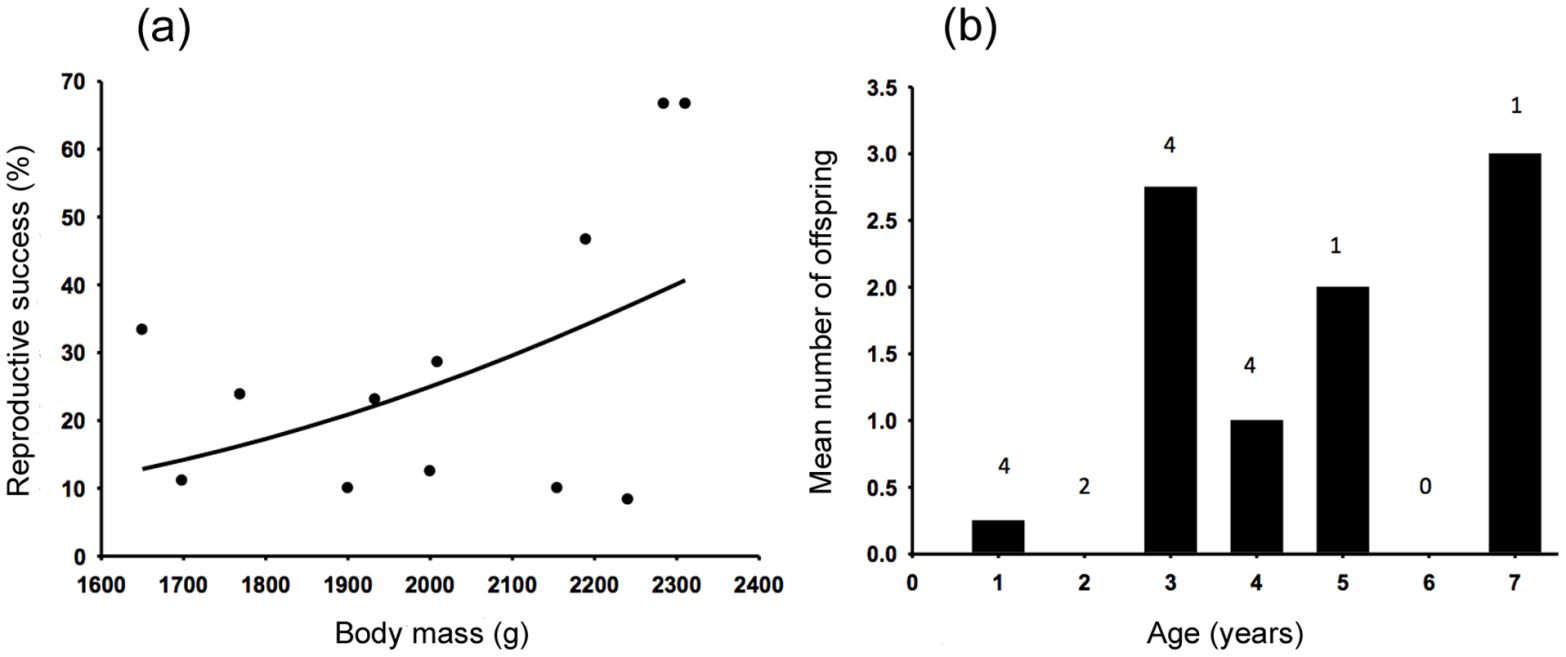
Relationships between male reproductive success and (a) body mass and (b) age class. (**a**) Relationship between the body mass of breeding male possums and reproductive success (%). The solid line indicates the logistic regression model, and (**b**) histogram showing the mean number of offspring fathered by males in each age class in 2005. Numbers indicate number of male possums in each age class.

Across all years, there was no significant difference between the mean body mass of breeding (mean ± s.e. = 2031±53 g, *n* = 15) and non-breeding males (2027±68 g, *t* = 0.04, d.f. = 30; *P* = 0.484; *n* = 17). However, among breeding males there was a significant positive relationship between mean body mass and reproductive success (deviance = 5.21, *P* = 0.022, *n* = 12; Fig. 4). There was no relationship between the number of females that a male had access to and his reproductive success (deviance = 1.03, *P* = 0.309, *n* = 20).

There was no significant difference between the age of breeding and non-breeding males in 2005 (*t* = 1.97; d.f. = 14; *P* = 0.068). However, the distribution of paternities across age classes was uneven. Three-year-old males fathered the most offspring, accounting for 52.4% of the 21 PY genotyped in 2005 (Fig. 4). The two most reproductively successful males were both three years old: one sired four and the other sired five offspring. The other two males in this age class each sired a single offspring. The next most successful male was a seven year old, who produced three offspring. Nevertheless, we had insufficient data to fully investigate age-specific reproductive success or the significance of the peak in the three-year age class.

## Discussion

Our results confirm a polygynous mating system in this population of *T*. *vulpecula* in a tropical *Eucalyptus* woodland. However, the life history traits of males differed from temperate populations in Australia and New Zealand [15], [16]. Reproductive success was highly skewed, with young, large males fathering the most offspring, which is consistent with results from another tropical population [17]. Our population experienced intense predation pressure from pythons, which was strongly biased towards male possums. We suggest that this may explain the earlier investment in reproductive effort by males, compared to possums in temperate regions.

We observed an unusually high rate of predation by pythons compared to other studies of arboreal marsupials [16], [27]. Over a two-year period pythons ate 60% of radio-collared males, and almost 40% of all radio-collared possums. In contrast, Clinchy [28] reported relatively low rates of predation in a population of *T*. *vulpecula* in southern Australia, with only 10% of radio-collared females being recorded as killed by a predator over a similar time period. Of these deaths, only one was attributed to a python, whereas an owl and a cat were responsible for one each, and five possums were believed to have been killed by dingoes or dogs. Similarly, a study of carpet python diets at four sites in the southern part of Western Australia recorded only one occurrence of a brushtail possum [29], while studies in eastern Australia have also found that brushtail possums form a relatively small proportion of python diets [30], [31]. Predation by pythons on *T*. *vulpecula* is likely to be more intense in the tropics, due to the smaller body size of possums (approx. 1500–2500 g compared to 2000–4500 g in southern Australia [14]); and the larger size of the snakes [32]. Furthermore, pythons are more abundant and active for a greater part of the year in the tropics [32]. Although pythons are active both on the ground and in trees [32], we only observed them on the ground at our site, including one observation of an ambush attack of a possum on the ground.

The strong bias in predation risk towards males is presumably a consequence of their larger home ranges and greater activity, compared to females. In the open eucalypt woodlands and savannas of northern Australia, males must spend significant amounts of time moving on the ground, as the canopy is discontinuous, thus exposing them to greater risk of predation by ground-dwelling predators. Furthermore, male possums have a large sternal gland which releases scent [14] and potentially allows pythons, which are ambush predators, to identify regularly used trees, den sites and pathways. The majority of predation events occurred in the wet season (December-March) of 2004/05, when we did not capture or observe any northern brown bandicoots, (*Isoodon macrourus* Gould; body mass 500–3100 g [33]). In contrast, after the 2005 dry season, we regularly caught bandicoots in our traps. Bandicoot abundance fluctuates substantially between years [34]. It is possible that in years when bandicoot abundance is high, pythons prey on them, but switch their diet to other prey items, including possums, when bandicoot densities are low.

Female *T. vulpecula* established solitary home ranges that generally did not overlap with other females, while home ranges of males were larger, overlapping the home ranges of between 2–10 females, and consequently other males. This pattern is common among polygynous mammal species [5], [35] and presumably reflects the males’ attempts to gain access to multiple females. We found that reproductively successful males restricted their mating to females whose home ranges they overlapped. Similar patterns were observed in *T*. *vulpecula* in southern Australia [16], as well as in studies of other mammal species, such as banner-tailed kangaroo rats (*Dipodomys spectabilis*) [36] and white rhinoceros (*Ceratotherium simum simum*) [37]. Similarly, only males that were captured repeatedly on the grid were reproductively successful, while we found no evidence that transient males fathered offspring.

The majority of females that bred multiple times mated with the same male at least twice and a large proportion only ever mated with one male. In contrast, all but one of the males who achieved multiple paternities mated with more than one female and many mated with multiple females within the same breeding season. The maximum number of females that any individual male mated with was four, and all of these females had home ranges at least partly overlapping his own. Consequently, about one-third of sibling pairs were full-sibling relationships. In comparison, Taylor et al. [15] found that in a population of *T*. *vulpecula* in New Zealand, only 15–20% off offspring were full-siblings. This suggests that males in our study population employ a relatively conservative mating strategy, establishing long-term home ranges from which they compete with neighboring males for access to a limited number of adjacent females. This strategy may indicate a trade-off between maximizing mating opportunities and predator avoidance in this population, with males seeking to limit the amount of time spent travelling on the ground to mate with multiple females.

Winter [38] suggested that male *T*. *vulpecula* form temporary monogamous pairs with females for 30–40 days before and during mating. This “consortship” was hypothesized to be a strategy of mate defense. We found that the interval between mating by individual males within a season was highly variable, and in many instances there was no opportunity for males to establish a consort relationship (mean interval = 28.53; s.d. = 20.12; minimum interval = 4 days; maximum interval = 57 days). Studies in New Zealand [15], [39], [40] have shown that the interval between successive matings by individual males was also generally shorter than suggested by Winter [38], indicating that those possums did not consort. It has been suggested that this difference in behavior may be a consequence of density, as temporary pair-bonding is more characteristic of solitary animals in low-density populations that have a reduced likelihood of contact [15]. However, it is also possible that in this population, males reduce the amount of time that they invest in mate seeking behavior, including consortship, in order to minimize predation risk.

Male reproductive success was highly skewed, with more than half of individuals not reproducing during the study, whereas a few fathered a large proportion of offspring. This finding is consistent with other studies in Australia and New Zealand [15], [16], [39], but the mechanisms driving these patterns appears to differ between populations. Although body mass did not determine whether a male bred or not, among those that did breed, reproductive success was positively correlated with body mass. Similarly, age had a significant impact on breeding success, with males in the three-year old age class producing the most offspring, with the exception of a single successful older male. Clinchy et al. [16] reported a positive relationship between body mass and paternity in male *T*. *vulpecula* in south-eastern Australia, but found that older males (eight years of age) were the most reproductively successful. In contrast, Taylor et al. [15] found no effects of either age or size on male reproductive success; however this may have been because that study was conducted on a population subject to culling. Our results are more consistent with those of Kerr [17], who similarly found that younger, larger males had a higher degree of reproductive success, although that study also reported a smaller peak in reproduction among older age classes.

Males in this population became sexually mature at 1–2 years of age, which is consistent with observations from another tropical population in the Northern Territory [41], although male possums on Magnetic Island near Townsville bred slightly later, reaching maturity at 2–3 years [17]. In comparison, studies near Armidale in southern Australia and in New Zealand both reported that male possums reached sexual maturity later, at 2–4 years [16], [42]. The interaction between sex and age had a significant positive effect on body mass, which indicates that males grew faster than females across this age range. This is consistent with the observation of increased reproductive investment in younger age classes by males. However, our analysis of the interaction between sex and age suggests that males were heavier than were females in both the 3-year-old and 4-year-old age classes, which includes their peak reproductive period, and thus these are the age classes when greater size is likely to be most advantageous.

The patterns of male reproductive success that we observed are most similar to those reported from tropical Magnetic Island, where younger, heavier males were also the most successful breeders [17]. This suggests that in tropical populations where the life-histories of *T*. *vulpecula* may be shaped by native predators, males invest in reproductive effort earlier in life, followed by another surge in reproductive effort in old age. Pythons and hence python predation on *T*. *vulpecula* are relatively uncommon on Magnetic Island [43], most likely as a result of insufficient smaller-sized prey items to sustain the transition from juvenile to adult snakes (Simon Fearn, pers. comm.). Nevertheless, the relatively recent shared evolutionary history of mainland and island possums in north Queensland supports the existence of similar life history strategies in *T*. *vulpecula*.

We demonstrated strong sex-bias in predation on this population of *T*. *vulpecula* in northern Australia, which has not previously been shown in other populations in temperate regions. To our knowledge, this is the first demonstration of sex-biased predation in an Australian marsupial. We also found clear differences in male life history traits in possums in northern compared to southern Australia. These results support our hypothesis that in populations of possums experiencing high predation pressure, male possums can adjust their mating tactics and reach sexual maturity at an earlier age. In this population, we also found that younger males were more reproductively successful than were older males; however, based on our data, we were unable to conclude that this was the result of an evolved strategy towards increased reproductive effort by younger individuals. Rather, it is likely to be the case that younger males do not face competition from older age classes and thus, their success is the result of increased opportunity. Nevertheless, it is clear from this study that increased predation risk in the tropics imposes a trade-off in male reproductive strategies between maximizing mating opportunities and avoiding predation in a population of marsupial folivores, which has not previously been shown in temperate populations.

Because of the high degree of sex bias and temporal variation in the intensity of predation, we do not assume that this population is regulated from the top-down. Conversely, we have previously demonstrated that female reproductive success in this population is limited from the bottom-up, through spatial variation in foliar available N concentrations among individuals’ home ranges [18]. Nevertheless, our study provides a convincing demonstration of differential impacts of top-down and bottom-up factors impacting simultaneously on the life history traits of males and females within a single population of mammals.
